# An Analysis of Students' Perceptions of Strategies to Improve Well‐Being in Dentistry

**DOI:** 10.1111/eje.13065

**Published:** 2025-01-09

**Authors:** Charlotte Cheuk Kwan Chan, Elise Hoi Wan Fok, Michael George Botelho

**Affiliations:** ^1^ Faculty of Dentistry The University of Hong Kong Hong Kong SAR China

**Keywords:** dental education, dental school stress, dental student, qualitative study, stressors, well‐being

## Abstract

**Introduction:**

A number of papers have reported on stressors to students in the dental curriculum. This paper analyses perceptions of strategies to improve well‐being among final‐year dental students in a dental curriculum.

**Methods:**

A literature review was performed to create a question guide to explore issues of wellness and stress in a dental curriculum. Final‐year dental students were invited to an interview using random sampling and issues related to strategies for well‐being were analysed by an inductive–deductive approach.

**Results:**

Fourteen interviews were conducted, yielding three themes under the overarching domain of strategies to improve dental student well‐being. Under the theme of well‐being management, students wished for training on stress reduction for their personal well‐being and guidance on communication, referral and mental health support to manage the well‐being of colleagues and patients. The second theme, mentoring, covered peer support in the form of a ‘buddy system’ and sharing from recent graduates to help students gain practical and career advice about post‐graduation challenges. Finally, suggestions for institutional support included providing in‐house counsellors in the dental hospital with specialised knowledge about the unique concerns of dental students and clear leave of absence policies that treat mental and physical health equally to encourage help‐seeking and reduce the fear of disclosure.

**Conclusion:**

The experiences of final‐year dental students were sampled to explore potential approaches to improve well‐being in the dental school environment. Guided by these student perspectives, specific strategies have been implemented and recommended to improve the wellness support provided by the faculty for dental students.

## 1 Introduction

Dentistry is a highly stressful subject, with a multitude of studies reporting poor levels of well‐being among dental students [1, 2]. Dental students often experience greater symptoms of burnout, depression and anxiety compared to students from other faculties, including medical students [3, 4]. This has been attributed to the challenging learning curve of acquiring psychomotor skills as well as the risks and unpredictability inherent in their clinical training [5]. A previous study in our institution revealed poor well‐being in the undergraduate dental student population, which worsened with increasing years of clinical study [6]. Less than a fifth of dental students felt that the institution had supported their wellness, with fear of failing exams, sleep deprivation and insufficient free time to pursue extracurricular interests cited as the main impediments to maintaining well‐being [6]. It has also been reported that healthcare students face barriers to seeking help for mental health challenges due to doubts surrounding the confidentiality of such disclosures, unfavourable ramifications for their studies and future career, as well as perceived stigma from faculty and peers [7, 8].

Low student well‐being and high stress levels are detrimental to students' physical health [9], enjoyment of the course, learning outcomes [10, 11] and patient care [12]. Despite the abundance of literature on the challenges of dental training and its adverse impacts, little research exists from a student perspective about how these stressors can be mitigated. In addition, healthcare students have expressed deficits in willingness, readiness and competency when it comes to managing patients affected by mental illness [13, 14]. This must be addressed through the provision of additional training in the curriculum, given the prevalence of such conditions in the general population. Moreover, the growing recognition of the importance of well‐being in the professional responsibility of dentists has led to its inclusion in the core competencies expected of graduating practitioners in international guidelines [15, 16].

The present paper forms the second of a two‐part qualitative analysis of final‐year undergraduate dental student well‐being. The first part analysed students' perceptions and experiences of stressors and well‐being in themselves, their peers and their patients. It also explored the themes of stigma and receiving and providing support at peer, faculty and professional levels. The aim of this second part is to highlight students' views on strategies to improve dental student well‐being.

The insights gained from this research can inform the development of curricular interventions to improve students' ability to recognise and address their own well‐being needs, as well as those of their colleagues and future patients.

## 2 Materials and Methods

This study adheres to the recommended guidelines outlined in the Consolidated Criteria for Reporting Qualitative Research (COREQ) (Appendix A) [17]. The study protocol was approved by the Institutional Review Board of The University of Hong Kong/Hospital Authority Hong Kong West Cluster (HKU/HA HKW IRB, Reference number: UW 21‐244).

To develop the interview guide, a literature review of existing quantitative and qualitative studies exploring the well‐being experiences of healthcare students was carried out. A total of 13 studies were selected to guide the question development process (Table 1) [7, 18, 19, 20, 21, 22, 23, 24, 25, 26, 27, 28, 29]. The interview questions were designed to elicit information in a neutral, open‐ended manner to address key topics under each of the three overarching domains of self, peers and patients.

**TABLE 1:** Question guide used during interviews and associated reference sources.

**Domain 1 and references:** Understanding final‐year BDS students' stressors and well‐being [18, 19, 20, 21, 22, 23]
**1.3.2 Do you feel that the BDS course has given you adequate training to manage your own well‐being?**
*YES → What aspects of the BDS course and how was it helpful?*
**1.3.2 What strategies by the faculty do you think should be considered to improve dental student well‐being?** *May discuss*: *changes in the course structure* *flexible attendance policies for mental health leave* *in‐house counsellors at PPDH* *survival guide* *workshops from CEDARS/NGOs on well‐being* *sharing from practicing dentists/recent graduates* *mentoring/buddy system* *more support from staff/personal tutors* (*give details*)
**1.4.1 Do you think there should be more topics integrated into the BDS curriculum about well‐being?**
*YES → What kinds of topics should students learn about well‐being? And how?*
**Domain 2 and references:** Understanding BDS students' perceptions and experiences of supporting mental well‐being of colleagues [7, 19, 20, 21, 23, 24, 25, 26, 27].
**2.4.1 Do you feel that the BDS course has given you adequate training to support colleagues with their mental well‐being?**
*YES → What training did you receive? What kind of learning topics or training would you like more of?*
*NO → What kind of learning topics or training would you have liked to have?*
**Domain 3 and references:** Understanding BDS students' perceptions and experiences of supporting mental well‐being of patients [24, 28, 29].
**3.3.3 Do you feel like the BDS training adequately covers treating dental patients with mental illness?**
*YES → Why?*
*NO → Why not?*
**3.4.1 Would you find training on supporting the mental well‐being of patients helpful?**
*YES → What kind of topics would you like such training to cover?*
*NO → Why not?*
**Other Open‐Ended Questions**
Before we end the interview, do you have anything else to add?

*Note:* Full interview guide available in Appendix B.

The interview guide consisted of 33 questions and was piloted with one of the authors (M.G.B.) and two final‐year students (C.C.K.C. and E.H.W.F.) to assess clarity and scope. After refinement, pilot interviews were undertaken with two fifth‐year students. The pilot test recordings were reviewed with M.G.B., an experienced qualitative researcher, as a calibration and coaching exercise to provide feedback on the interviewers' skills.

### 2.1 Participant Recruitment

At the start of the 6‐year Bachelor of Dental Surgery (BDS) programme, which consists of two preclinical years (Years 1–2) and four clinical years (Years 3–6) involving direct multidisciplinary patient care, students are randomly assigned by the faculty into seven clinical groups. To recruit participants for the study, the research team obtained a student list for each clinical group in the Class of 2024 and arranged the names of the 73 students in alphabetical order. An online random sequence generator was then used to randomly select two students from each group who were invited to participate. Informed consent was obtained from all participants, who were assured that their voluntary participation and any responses provided would not affect their status within the BDS programme.

### 2.2 Research Team

The research team consisted of two final‐year dental students, C.C.K.C and E.H.W.F., whose first‐hand experiences of the BDS program and clinical training guided the interview process and subsequent interpretation of the data. They were supported by a clinical professor, M.G.B., with extensive experience in teaching undergraduate dental students, curriculum development and qualitative research. Given the sensitive nature of the topics explored, all interviews were conducted confidentially by C.C.K.C and E.H.W.F. to create an environment that encouraged open and honest peer‐to‐peer sharing, without any influence or pressure from faculty staff. Anonymity and blindness were strictly maintained, with the audio recordings of the interviews accessible exclusively to the two student researchers, C.C.K.C and E.H.W.F.

### 2.3 Data Collection

All participants provided written consent to take part in the study. The interviews were conducted by one of two trained female interviewers, who were final‐year dental students (C.C.K.C and E.H.W.F.) and known to the participants.

Each interview lasted between 45 and 75 min and was done in English via Zoom (Zoom Video Communications Inc., Delaware, USA). Participants were encouraged to find a quiet and private space to attend the virtual interviews, which were scheduled in the evenings or on weekends, after their clinical sessions. Apart from the participant and the assigned interviewer, no other individuals were present during the interviews.

Interviews were guided by a semi‐structured approach with the interview guide of open‐ended questions as described in Part 1 of this analysis. The questions relevant to Part 2, ‘strategies to improve well‐being’, are shown in Table 1, and the full interview guide is available in Appendix B. Aside from utilising the prepared interview guide, the interviewers asked follow‐up questions as needed to elicit further information from the participants. All discussions were audio‐recorded using Zoom Version 5.17.11 and then transcribed verbatim. The transcripts were subsequently verified by the interviewers to ensure accuracy. Data saturation was reached after the fourteenth interview, after which no further participants were recruited and no repeat interviews were conducted. During the transcription process, all personal identifying information was removed to maintain the anonymity and blindness of the participants.

### 2.4 Thematic Analysis

The interview data were analysed using a thematic content analysis approach with Quirkos Version 2.5.3 software (Quirkos, Edinburgh, UK). The process involved data familiarisation and line‐by‐line coding performed by the two researchers (C.C.K.C and E.H.W.F.), through a dual analysis approach. This analysis was then reviewed with the clinical professor, M.G.B., to confirm the findings and resolve any differences in interpretation. Key themes and subthemes emerging from the data [30, 31] were identified with an inductive–deductive approach. C.C.K.C and E.H.W.F. initially conducted independent open coding on three data sets and then discussed these reflexively with M.G.B. This iterative process was repeated over several meetings until all data sets had been thoroughly discussed, and a consensus on the interpretation was reached. Participants did not provide feedback on the findings.

The related codes were subsequently clustered and organised into analytical categories, leading to the development of themes and subthemes. These were derived either directly from the participants' own words or through the interpretation of the research team. Throughout the coding and analysis process, the researchers made a conscious effort to avoid being governed by their own pre‐structured understanding and maintained a self‐reflective attitude. This allowed them to consider alternative interpretations and potential influences on the coding process during their open discussions.

## 3 Results

Seventeen final‐year BDS students were invited to participate in interviews, of which three declined; the first expressed a lack of interest and the latter two did not feel comfortable sharing personal experiences on this topic. Interviews with 14 students were performed until data saturation was reached. From the thematic content analysis, five domains were identified. Under the first four domains (‘awareness and understanding’, ‘support’, ‘stressors in BDS’ and ‘stigma’), 22 themes were identified and have been separately reported in Part 1 of this analysis (reference to be added later—paper currently in submission). The final domain, ‘strategies to improve well‐being’, yielded three themes (‘well‐being management’, ‘mentoring’ and ‘institutional support’) and seven sub‐themes as shown in Table 2.

**TABLE 2:** Domains, themes and subthemes.

Domains	Themes and subthemes
1. Awareness and understanding	1.1 Signs and symptoms of individual wellbeing
	1.2 Signs and symptoms of wellbeing of peers
	1.3 Mental health of patients
	1.4 Professional responsibility
2. Support	2.1 Receiving peer support
	2.2 Providing peer support
	2.3 Faculty‐level support
	2.4 Professional support
	2.5 Other support
3. Stressors in BDS	3.1 Curricular stress
	3.2 Transitional stress
	3.3 Clinical stress
	3.4 Interpersonal stress
4. Stigma	4.1 Internal stigma
	4.2 External stigma
5. Strategies to improve well‐being	5.1 Well‐being management
	5.1.1 Training on managing personal well‐being
	5.1.2 Training on managing well‐being of colleagues
	5.1.3 Training on managing well‐being of patients
	5.2 Mentorship
	5.2.1 Peer mentorship
	5.2.2 Sharing from dentists and recent graduates
	5.3 Institutional support
	5.3.1 In‐house counsellors
	5.3.2 Flexible attendance policies

*Note:* Domains 1–4 were previously reported in a separate paper.

### 3.1 Theme 1: Well‐being Management

#### 3.1.1 Training on Managing Personal Well‐being

None of the interviewed students felt that the faculty had provided ‘adequate training to manage [their] well‐being’. Half of the students (50%) would find the integration of well‐being topics into the BDS curriculum helpful (P1, P2, P3, P4, P5, P6 and P14), particularly after the first year of the course: ‘would be helpful if they at least kind of talked about … at least once’ (P6), ‘only in the first week of the BDS curriculum there is an orientation week … there is a lecture arranged by CEDARS about the mental health of BDS students, I think that's it’ (P4). Greater acknowledgement of well‐being in the curriculum would help students feel that it is taken seriously by the faculty: ‘From what I experienced in the past six years, there's just nothing about well‐being. Like, it's not even talked about, it's not ever discussed … I feel like it's just not a part of the curriculum. They aren't concerned about it at all’ (P12).

With regards to the content of personal well‐being topics, several students suggested covering the skill of stress management in the curriculum: ‘how to deal with stresses’ (P3), ‘general stress management’ (P1), ‘managing their own feelings like when there are some anxiety or challenges … some coping measures’ (P2), ‘there could be some seminars … sharing some stress reduction strategies’ (P14). Two students commented on the heavy academic focus of the BDS course and expressed a desire for specific sessions to be focused on stress relief and relaxation: ‘we only focus on academic stuff, but we do not have any like sessions to help us relax’ (P7), ‘how to promote your well‐being non‐academically … enjoying things outside of dentistry, that would also be helpful’ (P1).

On the other hand, some students also expressed concerns that the introduction of mandatory wellness‐related courses into the BDS curriculum would not be well received by students: ‘I also don't see BDS students being receptive to like wellness courses’ (P6), ‘not sure if it will be like, taken that seriously if it is integrated into the curriculum’ (P1), ‘I'm just afraid that if integrated in the curriculum … then there will be more lectures and also more stuff to be examined … we don't want more lectures’ (P13).

Contrasting this, many students felt that learning to manage individual well‐being could be directly taught and that students needed to develop resilience independently through overcoming challenges and gaining experience. ‘I think the experience of having gone through the course equipped me with the experience to manage it, not really like the course itself. There's nothing we learn in particular that would help us. I guess it's kind of all just you learn as you go’ (P6), ‘I don't think the faculty itself has much responsibility to teach about well‐being, but I think the responsibility is there in the sense that there should be support to students when it's needed’ (P9), ‘don't think the BDS course can have any lessons or other curriculum that can improve our well‐being’ (P10), ‘I think it's mostly learning on our own. If we're having classes, people might not listen, and listening is not the best way to learn in my own opinion, you have to experience that yourself and then you can learn a lot faster … we need time to learn, but our course is long enough to have time to like, get to know how to deal with different problems and solve it on our own’ (P11).

#### 3.1.2 Training on Managing Well‐being of Colleagues

Students were more receptive to receiving training on managing the well‐being of their colleagues, with over 70% of students finding this helpful (P1, P2, P3, P4, P5, P6, P8, P10, P11 and P12). When asked specifically about the content of such training, students wanted to ‘learn about some things we can do practically to help colleagues with mental well‐being’ (P5), such as providing ‘verbal support’ (P13) and ‘how to counsel them’ (P10), as well as the identification of problems and their subsequent management: ‘how to detect signs of mental illness, and what can you do when you detect your colleague is having it’ (P3). A variety of formats were suggested for the delivery of this training, including ‘small group meeting’ (P3 and P2), ‘role play’ (P10), ‘workshops’ (P6 and P10) or sharing from ‘psychologist or counsellors’ (P4).

It was suggested that this training could be provided on a voluntary basis for students. ‘If it is compulsory then some of the classmates may feel like it's just unnecessary and wasting their time’ (P14), ‘giving a class … may give more stress to the BDS students, so I think it can be like a voluntary lecture for those who are interested, or a leaflet or poster, or Instagram post, then you can refer to it when you need that’ (P13).

#### 3.1.3 Training on Managing Well‐being of Patients

All students desired increased training on managing the well‐being of patients, including content on patient communication and understanding the oral health implications of mental illness. Students recognised that mental illness was often viewed separately from physical illness: ‘we're mostly trained on how to deal with medically compromised patient, like heart disease, hypertension, DM, etc., but not on mental health patients’ (P7), ‘the mental health part is integral to the patient … we see that blood pressure is part of the patient, so we keep this in mind and manage it, but for mental illness it is unmentioned’ (P14). Students wanted to learn more about ‘common mental illnesses’ (P1), such as anxiety, depression or ‘dental phobia’ (P1), as well as other less common conditions [‘schizophrenia or psychosis … PTSD’ (P6)]. In addition, they wanted to learn ‘strategies to improve my treatment for patients with mental illness’ (P5), ‘how to provide an environment that is welcoming’ (P14) and ‘when to refer’ patients to psychological help (P7 and P10).

Interestingly, several students brought up the importance of learning to support patients with mental illness in light of the widespread prevalence of mental disorders in society (P1, P7, P9 and P14), and two acknowledged the challenges posed by the stigma of disclosure. Participant 1 suggested learning how to ‘manage patients who may not disclose things to you’ due to ‘stigma’. Participant 7 shared: ‘Hong Kong is a very stressful city, and I think there are a lot of people with mental illness of different degrees … perhaps there may be some special skills to deal with … those patients [who] said they are not diagnosed with mental illness, but they have some sign of mental illness that we can observe’.

As students were generally already well aware of signs and symptoms of mental disorders, they wished for ‘practical’ (P8 and P13), ‘detailed tips’ (P8) on patient management and communication. ‘We do know about the illness itself, and what it is, and how what type of symptoms might be there, but I don't think we get much of a realistic patient to dentist experience … I think they could give us a dialogue of things to say or not to say in front of a patient with anxiety, for example’ (P8), ‘tell us what are some helpful and unhelpful things to say’ (P14), ‘the way we should talk to patients with mental illness is not taught’ (P11). Students were worried about upsetting the patient with insensitive communication: ‘patients with mental illnesses … might easily be triggered with, like some languages from the dentists’ (P11), ‘might be quite sensitive to like certain words … I do think that it's a responsibility that we don't make them feel like discriminated or uncomfortable’ (P12).

With regards to the format of such training, students proposed a range of suggestions, with sharing from experts such as psychologists or psychiatrists (P2, P12 and P14) and ‘role play’ or ‘workshops’ (P10, P11, P12 and P14) favoured over ‘lectures’ (P4 and P12) and ‘PBL’ [problem‐based learning] (P12).

Given that not all students are guaranteed exposure to patients with mental illness in their BDS training, two students suggested including more patients with mental health needs in the teaching patient pool: ‘The exposure is quite limited … could be helpful, especially if we get more patients with mental illnesses in in school … it would help a lot with learning about mental illness and how to adapt like my treatment to their specific needs’ (P5), ‘there are really some practical, like, true experience with dealing with patients with mental illness … we need to really meet different patients with like certain kinds of mental illness, then we will grow and learn how to deal with them’ (P13).

### 3.2 Theme 2: Mentorship

#### 3.2.1 Peer Mentorship

Ninety‐three percent of students would find ‘peer mentorship’ or a ‘buddy system’ helpful for their well‐being (P1, P2, P3, P4, P5, P6, P7, P8, P9, P11, P12, P13 and P14). As discussed in Part 1 of this paper, most students preferred to turn to their peers for support over faculty staff or mental health professionals, as they believed that other dental students would be able to better understand and empathise with them through shared knowledge and experience of the stressors. This was reiterated in their strong backing for a ‘mentor and mentee system so that [students] can share more of their personal experience’ (P3), which could provide ‘a kind of heads up … so that the incoming years can know what to expect … from the course and career path, so it's not as daunting’ (P9). Students felt that there was an existing social gap whereby some junior students had no connections to senior students: ‘the faculty didn't do much of a job where they set up a system where the senior could contact us, and we can contact the senior … some of us have seniors we know, so we can personally ask. But if you're someone who doesn't have much of a connection to the seniors, then you're really alone’ (P8).

Peer support was more readily received than tutor support, as they were perceived to be on equal footing. ‘A buddy will be better than the staff, because sometimes the staff is not on the same level as us’, P7; ‘easier for us to like talk to each other because we are in like a similar age group’ (P2), and have undergone similar experiences: ‘having someone who has gone through all of this and having someone who can empathise with that would actually be more helpful’ (P8), ‘the senior … they're still studying, they know the situation and they may give us some solution that they solve the problem that we had’ (P7), ‘will also be useful for the junior students, because we can offer a lot of our fresh experience to them … we just experience those struggles’ (P2).

With regards to the nature of peer mentorship, students suggested having a ‘group chat WhatsApp’ to enable students to ‘ask … whenever we have any troubles’ and facilitate discussions on ‘how to solve the stress or how to do things correctly’ (P8). Seniors could provide reassurance and reduce uncertainty ‘telling us that it is actually okay and talk more about the details … because usually the stress may be because of the unknown’ (P13). The mentorship would ideally involve senior students guiding junior students during the clinical transition period, which was identified as one of the most significant periods of stress during the programme: ‘it would be great if we have a senior to guide us when we freshly get into the clinic. I still remember when I was struggling … the senior taught me how to do it, I just felt that the world brightened in front of my eyes’ (P14).

The only student who was not in favour of peer mentorship (P10) felt that this could potentially worsen the existing stress of senior students: ‘In BDS curriculum usually the seniors have way more stress than the juniors. So, if the juniors have stress, and the senior also have stress, and then the junior finds the seniors, then there will be an overload of stress, and the senior will feel even more stressed. [Laughs]. But as a senior you cannot tell the junior you are stressed as well. So actually, having to manage the juniors' stress is adding to the seniors' stress as well, and adding to the workload’. Another challenge to a buddy system was the ‘really large gap’ in content and assessment styles between years due to changes and developments in course structure and design: ‘what the BDS 5 could offer might not really be applicable at the moment to the BDS 1’ (P6). Moreover, its effectiveness would ‘depend on the people themselves … if the senior is willing to reach out and be open and like create like an area of conversation … to feel comfortable’ (P6).

#### 3.2.2 Sharing From Dentists and Recent Graduates

Eighty‐six percent of students would find mentorship or sharing from more experienced dentists helpful (P1, P2, P3, P4, P5, P6, P7, P8, P9, P11, P13 and P14). Connections with a ‘practising dentist’ would be ‘more relevant for the higher years’ (P6), enabling senior students who may be serving as a ‘buddy’ for junior students to also receive mentorship themselves.

Students particularly valued the opportunity to hear from dentists working in private practice, where the majority of fresh graduates from this university would be employed after completing their BDS degree: ‘the mentoring from dental practitioners has also helped because it is different from tutors working in PPDH [university dental hospital] full time, it's more helpful to hear from those who are working in private practice, we can have a different insight of how things are going’ (P14). Practical advice about overcoming foreseeable post‐graduation challenges was welcome ‘because sometimes we will get stressful about the future, about how to plan our career, or even financial plans … we don't know much about that, so maybe sharing from the recent graduate will help’ (P4).

While students reported having ample opportunities to interact with experienced dentists throughout their course, these conversations were rarely of a personal or emotional nature: ‘we have it now, with current dentists, but I don't think we have shared about mental well‐being related issues’ (P3). Nevertheless, they would find it easier to open up to a dentist designated as their personal mentor compared to clinical tutors who taught or assessed them: ‘the personality of the mentor is welcoming and not judgmental’, whereas students may be ‘afraid to share some stuff with the clinical tutors’ (P14). The key barriers associated with seeking faculty support have been previously reported in Part 1 of this study.

### 3.3 Theme 3: Institutional Support

#### 3.3.1 In‐House Counsellors

Over half of students (57%) requested the provision of in‐house counselling staff at the dental hospital (P1, P2, P6, P7, P10, P11, P12 and P13). This would overcome the accessibility barriers of location and limited availability that have been attributed to the student counselling services (CEDARS) at the main university. ‘In‐house counsellor from PPDH is quite good … if they feel sad, they can directly go to the counsellors after the clinical sessions … it's also more time saving … you don't need to really travel’ (P13), ‘for some people who actually are having a hard time it's crucial to have someone to talk to, but the distance is definitely a problem cause we don't have time to travel that much, we don't even have time to go for lunch sometimes’ (P11).

Students highlighted the need for these in‐house counsellors to have knowledge specific to dental students with regards to their needs and concerns, building upon previous concerns about the overly generalised advice given by counselling staff at the main university which limited its usefulness. ‘The counsellor would be primarily … in contact with … dental students, so he or she needs to also be more skilled with handling, like our problems … kind of like more specialised’ (P12), ‘somebody who knows a bit more about the curriculum would help, because they'll be able to give a bit more concrete advice’ (P10).

A potential problem with providing in‐house counsellors is the stigma of being seen to utilise this service: ‘In‐house counsellors may or may not help, because if we are seen going to see the counsellors, I'm not sure how other people like students, nurse or teaching staff would feel’ (P14). This could be mitigated by providing support ‘online’ (P8 and P9), such as ‘an online Q&A with like the Google forms, or something, regarding any questions or struggles we have’ (P8).

While an in‐house counsellor may not be practical ‘because the faculty is so small’, Participant 10 suggested that the faculty could designate ‘somebody that we can turn to … if we want to speak to someone’ and students should be made aware ‘whom should we approach, and which tutors would be open to talking about our personal well‐being issues’.

#### 3.3.2 Flexible Attendance Policies

Students wished for greater leniency with taking time off school to look after their emotional well‐being and felt that ‘sick leave’ or ‘leave of absence’ should be extended to include ill‐health of both physical and mental nature, instead of only covering physical illnesses. More than half (57%) of the students would find these ‘attendance policies’ helpful (P1, P2, P6, P9, P10, P11, P12 and P14) but many noted that there were significant ‘miscommunications’ (P8) about the existing leave policies, which were not ‘clear’ (P14) and created stress and uncertainty.

However, students may potentially take advantage of such policies [‘if that happens, then a lot of people would be doing that every day’ (P7)] and it would be challenging to facilitate make‐up sessions for the lost clinical exposure once a leave of absence is taken [‘not easy to implement … students do need to make up for those missed practice … difficult for the faculty to organise the supplementary sessions’ (P2)].

## 4 Discussion

### 4.1 Theme 1: Well‐being Management

#### 4.1.1 Training on Managing Personal Patient Well‐being

As reported in Part 1 of this study, international guidelines on the expected competencies of dental practitioners have emphasised the professional importance of managing personal and inter‐personal well‐being in the dental team [15, 16]. In the 2023 FDI General Assembly, the World Dental Federation [32] advocated for dental educational institutions to provide accessible resources, including online counselling and helplines, for dental students struggling with their mental health and to equip students with the necessary knowledge and skills to look after their well‐being during their studies and future career. Increased recognition of the need to address dental student well‐being has emerged from recent reviews documenting burnout, stress and psychological ill‐health in the dental profession [30, 31], which have been associated with adverse outcomes on patient care and safety [33]. However, none of the interviewed students felt that they had received sufficient training to manage their well‐being and half would find the integration of well‐being topics into the curriculum ‘helpful’.

As several students reflected on the limited benefit of generic advice from university counsellors, training on personal well‐being for dental students should be contextualised to the specific challenges of the dental profession. This can be framed around a review of mental health and well‐being in dentistry commissioned by the UK's General Dental Council (GDC) [30]. They grouped the key stressors in dental practice under the domains of: business (e.g., finances, staffing and management), working environment (e.g., tensions with colleagues, heavy workload and time pressures), clinical (e.g., medically complex patients, difficult treatments, medical emergencies and clinical complications), patient‐led (e.g., complaints, litigation, managing patient expectations and dealing with challenging or dissatisfied patients), societal (e.g., regulatory and social pressure) and personal (e.g., perfectionism, self‐perception of skills and competence and feeling undervalued). As dental students are involved in clinical practice during their training, they are likely to encounter stressors identified in the GDC review aimed at dental professionals, such as handling clinical complications, engaging with challenging patients and grappling with personal stressors such as perfectionism and self‐doubt, making it highly relevant. By understanding these stressors early on, students will be better prepared to learn and develop effective coping strategies.

**FIGURE 1:**
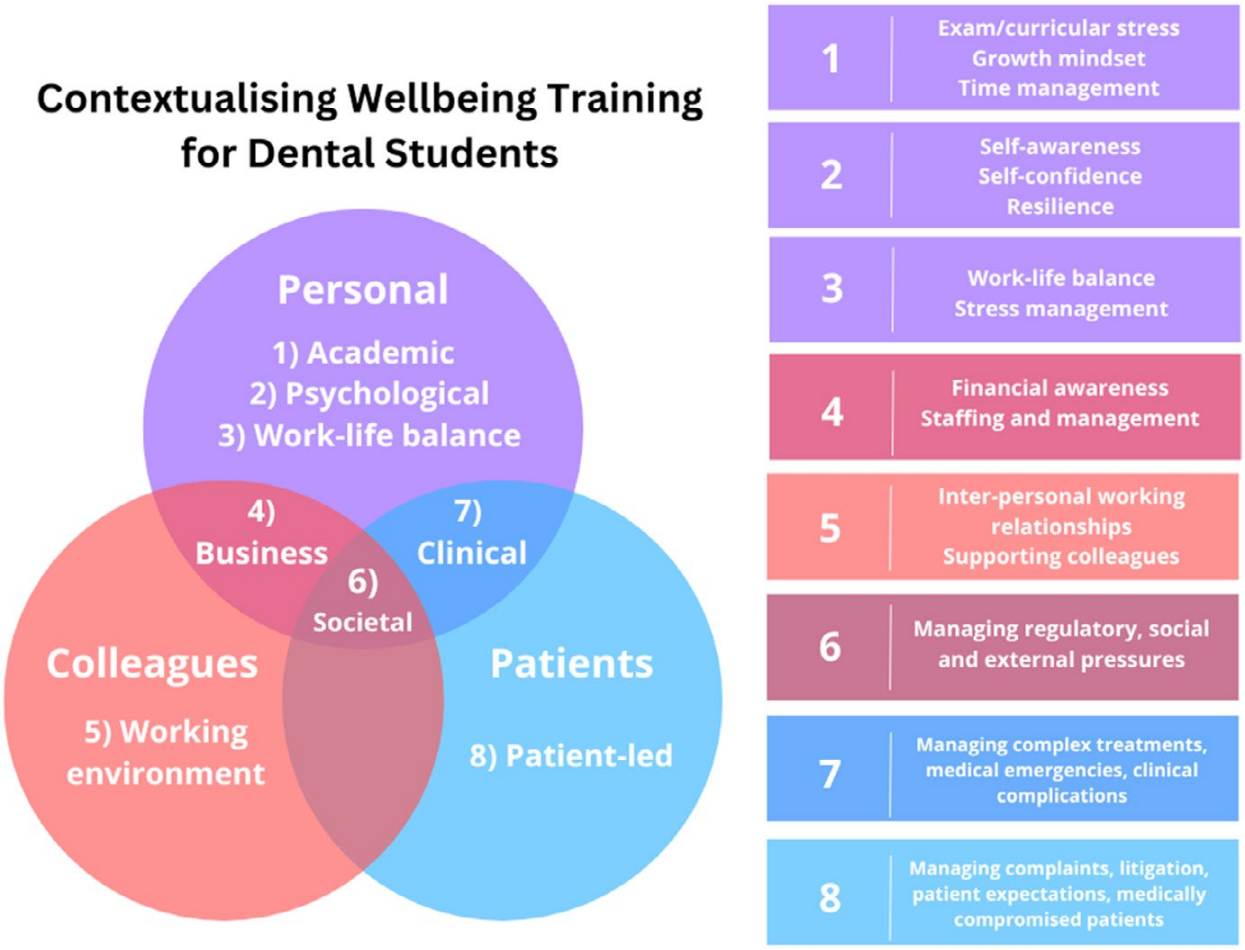
Contextualising well‐being training for dental students, based on findings from [1, 3, 6, 9, 30].

In addition, stressors among dental students have been previously reported from multiple global institutions, and most commonly involve examinations, fear of failure or falling behind, inconsistent feedback between tutors, lack of time for relaxation and a lack of confidence in one's abilities [1, 3, 6, 9, 30]. This was also reflected in the responses of the present study, with students requesting topics on ‘stress management’ and work–life balance ‘outside of dentistry’. These factors, representing actual or potential stressors that will be experienced during their studies and professional careers, need to be addressed when developing training to manage dental student well‐being and are presented in Figure 1. Educating students on these stressors may not address their immediate well‐being concerns as directly as the provision of direct counselling services, but nonetheless forms an important preventative approach to improve well‐being by equipping students with the necessary knowledge and awareness to manage the challenges they will inevitably face as qualified dentists.

The fact that a fifth of students were worried that incorporating well‐being training into the curriculum would increase their academic workload underscores the pressing need to effectively convey the critical importance of well‐being in professional responsibility and provide better stress management resources. While some students suggested providing well‐being training on a ‘voluntary basis’, such an arrangement could undermine efforts to destigmatise mental health within the broader student community, as concerns were expressed that being seen to utilise counselling services might elicit judgement from peers or staff. Integrating wellness topics across the curriculum for all students would not only convey that these issues are taken seriously by the faculty, but would also normalise conversations surrounding well‐being and ensure that all students are encouraged to acknowledge its importance.

With regards to the delivery of such training, students wished to obtain professional wellness management strategies from psychologists or psychiatrists, and preferred interactive formats such as ‘role play’ and ‘workshops’ over ‘lectures’. Two systematic reviews summarising stress management and well‐being initiatives in dental students [33, 34] have suggested online stress management programmes due to their cost‐effectiveness and ease of access, and have covered a range of techniques including yoga, breathing exercises, meditation, individual psychotherapy, cognitive behavioural therapy, life coaching and videos about imposter syndrome and procrastination. However, Plessas et al. [33] noted that all of the included interventions only dealt with the detection and management of prevailing stress and well‐being issues, with a paucity of primary prevention interventions eliminating inherent or structural sources of stress within the educational environment.

#### 4.1.2 Training on Managing Well‐being of Colleagues

Dental practitioners also have a responsibility to assist members in their workplace to maintain good health and well‐being both as a member of a team and team leader [16]. In preparation for this, dental students should be trained in basic peer support to recognise signs and symptoms of poor well‐being in others, and made aware of the available resources and opportunities for development to which they can refer their colleagues in need and has been initiated by a number of healthcare organisations. The American Medical Association has created a series of continuing education webinars on topics such as ‘Stress First Aid for Health Care Professionals’, ‘Building Well‐being into Culture’ and a ‘Wellness‐Centred Leadership Playbook’ to foster a supportive culture surrounding wellness in the medical team environment [35]. The Hong Kong Academy of Medicine, which includes the Hong Kong College of Dental Surgeons, has launched a Peer Support Scheme for fellows and specialist trainees, currently run by 48 peer supporters who have undergone specialised training by the Hong Kong College of Psychiatrists [36]. Similar peer support initiatives have been offered for dental professionals in the United States [37], Australia [38] and Canada [39]. Confidential hotlines offering emotional support have also been set up for dental professionals by professional associations [40] and indemnity providers [41], with equivalent services available for university students [42, 43].

#### 4.1.3 Training on Managing Well‐being of Patients

While the management of psychiatric conditions falls outside of the scope of a dental practitioner, dental students should be trained in appropriate referral of patients to psychiatric and psychological support. In addition, they should be taught about how oral health and well‐being are interlinked, including the oral health consequences associated with poor mental health and mental illness. Students expressed a desire for ‘workshops’ from mental health professionals on communication strategies to learn how to use appropriate, non‐judgemental and non‐stigmatising language when interacting with patients with mental health needs. Although two students suggested increasing exposure to patients with mental illness in their BDS clinical experience, this may not be feasible within the current curricula arrangements. However, exposure could be gained by providing dental outreach services in collaboration with non‐governmental organisations working with special needs populations, which has been previously conducted by this institution [44, 45]. In a UK dental school, an observational outreach programme provided undergraduate students with the opportunity to observe special care dentists in a mental health hospital and helped them learn ‘patience’, ‘communication’ and ‘empathy’, as well as the challenges in treating this population [46]. At another institution, the delivery of case‐based seminars about the dental management of individuals with psychiatric disorders was followed by a practical course for students to provide direct dental assisting for dentists treating this population at a social organisation. Although students had fewer negative emotions and more positive reactions to this population after the programme, their social acceptance and readiness to engage in treating them did not improve significantly [13].

### 4.2 Theme 2: Mentorship

#### 4.2.1 Peer Mentorship

Building upon the peer support discussed in the earlier section on supporting colleagues, peer mentorship was regarded as a ‘helpful’ strategy to improve dental student well‐being by 93% of respondents. Students gravitated towards seeking support from their peers over faculty staff, viewing their peers as more approachable and better equipped to empathise with the students' challenges, which has also been reported in past studies of peer mentorship in dentistry [47, 48]. To ensure the contemporaneity and relevance of the support given, and to overcome the challenge raised by one student (P6) about potential curriculum changes, mentors should be of proximate academic standing to mentees (e.g., pairing Year 1 students with students from Year 2 or 3, rather than Year 6).

A ‘buddy system’ has recently been piloted in our institution as a ‘Students as Partners’ project, pairing a group of Volunteer 21 senior year mentors to 49 third‐year mentees as they navigate the transition to clinical practice, which was identified as a major source of stress in the dental curriculum [6]. The mentorship included a clinical tour trial run organised by the senior students, involving demonstrations on record keeping, an overview of common dental instruments and procedures, and communication tips to patients, auxiliary staff and the dental laboratory. Participants shared positive feedback of the ‘friendships gained’, ‘easy implementation’ and ‘knowledge exchange’. In the coming academic year, this will be formally integrated into the third‐year clinical induction week. Other peer mentorship programmes for dental students have been reported by at least seven other dental schools in the United States [48, 49, 50, 51, 52] and Australia [47, 53]. A qualitative analysis of peer mentorship in dental students revealed that such programmes benefit both mentees and mentors by forming a sense of collegiality and kinship and can bring professional advantages by ‘training students to be comfortable with unfamiliar conversations and enhance their communication skills’ [47].

Only one student did not support peer mentorship (P10) due to the increasing burden or stress for senior peer mentors. This reinforces the need to provide stress management and wellness training for all students at an early stage in the curriculum, as well‐being in dental students has been shown to decrease across their training in longitudinal studies [54, 55]. Despite the benefits of mentorship stated earlier, if senior students manage their mental well‐being poorly, negative emotions such as stress, anxiety, dread or a pessimistic outlook could also be reinforced or passed down to younger students through the intimate mentor–mentee relationship.

#### 4.2.2 Sharing From Dentists and Recent Graduates

Similarly, a large majority of students (86%) reported mentorship or sharing from more experienced dentists helpful, especially as a senior student. At our institution, the latter has been incorporated into a series of ‘Preparing for Dental Practice’ workshops offered to final‐year students to prepare them for the hands‐on, everyday aspects of independent clinical practice, where experienced dental practitioners are invited to share advice and their experiences of success and overcoming adversity with regards to clinical practice and business management. In addition, this institution and the local dental association currently co‐organise an annual mentorship programme connecting practising dentists with senior students. Students reported that ‘mentors were extremely enthusiastic’ (third‐year students), and some graduated students have even ‘become good friends or even business partners’ (mentor) with their mentors [56]. Students benefitted from the opportunity to ‘seek advice on our future path, possible clinic attachments in dentists' clinics and know more about the dentistry world outside the Faculty’ (fourth‐year student), and gained ‘invaluable insight’ about the ‘transition into the initial stages of our career’ (fifth‐year student) [57]. The pervasiveness of social media has enabled students to readily connect with alumni and practising dentists through various social media platforms and online discussion groups. Other active groups bringing together dental students and practising dentists through formal or informal mentorship include those organised by medical indemnity companies such as Dental Protection [56], professional associations like the British Dental Association's ‘Future Leaders Programme’ [58] and the Australian Dental Association [38], and study clubs run by dental companies [59, 60].

### 4.3 Theme 3: Institutional Support

#### 4.3.1 In‐House Counsellors

The provision of in‐house counsellors was favoured by over half of the interviewed students (57%) for reasons of greater ease of access and increased familiarity with the dental curriculum, which would enable more specialised and dedicated support. In contrast to the aforementioned strategies, which involved secondary level support through destigmatisation, social networks, training and psychoeducational interventions, in‐house counsellors are an example of tertiary‐level support, usually providing services to individuals already experiencing psychological needs [33]. Although most universities, including ours, offer counselling services to students, many are not tailored specifically to dental students' needs despite evidence of the value of contextualising psychological support to the dental education setting. A study in Chile offered dental students eight 45‐min weekly psychotherapy sessions to enhance their ability to manage dental environment‐related stressors, which reduced perceived stress and improved self‐reported coping skills among all participants [61]. Moreover, the participants who had displayed dysfunctional psychological scores prior to the intervention were able to return to the normal range after the therapy [61]. At a US dental school, which provided in‐house counselling, a positive relationship was found between the number of attended counselling appointments by students and their overall functioning, suggesting that the provision of such services helped meet their needs. Students' presenting psychological concerns centred around academic pressure, time management, exam anxiety, low self‐esteem, interpersonal conflicts, substance abuse, eating/body image concerns, work–life balance and financial issues. To protect the confidentiality of students seeking counselling, the centre was located in an area of low foot‐traffic in the dental building, and the psychologist reported to both the Associate Dean for Student Affairs in the dental school and the Director of the University Counselling Centre [62].

If resource limitations preclude the feasibility of employing in‐house counsellors, at a minimum, courses such as Mental Health First Aid should be provided to personal tutors and staff. This has now been undertaken by a number of staff at our institution. It is also important that students are aware of whom to approach when in need of support, since the first part of this study reported on how uncertainty could deter students from seeking help. This could be accomplished by introducing these staff members and the scope of their roles in student manuals.

#### 4.3.2 Flexible Attendance Policies

The interviews revealed that over half of students sought greater clarification and guidance on attendance policies and indicated that they would find it helpful if these policies included provisions to accommodate poor mental health. As some students raised concerns that such accommodations might be taken advantage of, there is a critical need to clearly define a ‘mental health day off’. At the same time, unfounded fears were expressed by UK medical students that disclosing mental illness in requesting accommodations would result in regulatory ‘fitness to practise’ proceedings that would directly lead to expulsion and reputational damage, which contributed to a detrimental culture of ‘presenteeism’, or the normalisation of attending clinics and classes when ill [27].

In forming attendance policies that accommodate mental health, dental institutions can refer to guidance published by medical schools and associations on this matter. In the United Kingdom, the junior doctor contract legally states that medical professionals are entitled to take time off work to address mental health concerns, in the same manner as they would for physical illness [63]. The General Medical Council released a 2021 report on ‘Supporting medical students with mental health conditions’, which asserts that ‘medical schools have a duty to make reasonable adjustments for students with long‐term mental health conditions, to help them to study medicine and meet graduate outcomes’ [64].

Canadian medical schools have published a document on ‘Personal Day Policies’, defining a ‘personal day’ as an ‘absence that medical students can take when they recognise they are experiencing burnout, anxiety, depression, and/or other indications that their mental health is suffering. A personal day is not necessarily defined by urgent mental health concerns and is viewed as an absence one can take to actively cope with the stressors of medical school and/or one's personal life. Furthermore, the scope of a personal day is not limited to physical or mental health. Personal days are inclusive of circumstances that may not be foreseeable (e.g., appointments to address financial concerns with the bank)’ [65]. At the University of Adelaide, medical students are permitted to take unplanned leave for mental health reasons, known as ‘mental health days’, with school policy explicitly stating that mental illness is treated in the same manner as physical illness and there is no limit on the amount of absence taken [66].

### 4.4 Strengths and Limitations

The strengths of the present study include its qualitative nature, facilitating a deeper exploration of dental students' views and perceptions of strategies to improve their well‐being, which was guided by a question framework developed from past quantitative research [6]. Our target participants were final‐year students, who may be more likely to provide honest sharing out of motivation to improve the student experiences of future dental cohorts. Moreover, their imminent graduation may reduce concerns about how their responses might be perceived by the faculty. To avoid self‐selection bias, random sampling was used and 17 students were invited, with 14 agreeing to participate. The obtained sample was demographically representative of the final‐year BDS cohort in terms of gender, nationality and age, and the COREQ (Appendix A) guidelines were followed to minimise reporting bias.

Although the interviewers were known to the participants, participant anonymity was ensured. It was believed that students might be more willing to open up to peers of similar background and age, compared to staff members, who may have been associated with a power differential. The interviewers, who were also final‐year dental students, may have inadvertently introduced bias into the research process through their personal beliefs, assumptions and experiences. However, this was mitigated as much as possible by maintaining reflexivity through frequent triangulation with an academic staff member and co‐author. The present sample was drawn from a single institution, so the findings may have limited extrapolation to other dental schools with different curricula.

## 5 Conclusion

This qualitative study explored potential strategies to enhance dental student well‐being. Regarding training, students expressed a desire for content on stress management to improve their personal well‐being. Additionally, training on supporting the well‐being of others should include recognising signs and symptoms of poor mental health, communicating sensitively and empathetically, and understanding when and how to refer individuals to professional support.

Peer mentorship emerged as a popular approach to improve social connections and reduce fears and uncertainties, by providing junior students with an experienced senior mentor for reassurance and guidance. For final‐year students, mentorship from practising dentists or recent graduates could help support their career planning and transition to the workplace.

To potentially improve the accessibility and relevance of counselling support, dental schools could consider providing in‐house counsellors with specialised training or experience in dentistry‐related issues. Alternatively, designating faculty members with mental health first aid training to serve as initial points of contact for students may reduce barriers to help‐seeking.

To provide students with the structural support necessary to manage and address poor personal well‐being, and to prevent more serious mental health crises or the need for extended long‐term leave, schools should consider developing clear leave of absence policies and reasonable adjustments that treat mental and physical health issues with the same degree of seriousness and importance.

## Conflicts of Interest

The authors declare no conflicts of interest.

## Data Availability

The data that support the findings of this study are available upon reasonable request from the corresponding author.

## Appendix A

### Consolidated Criteria for Reporting Qualitative Studies (COREQ): 32‐Item Checklist

Developed from: Tong A, Sainsbury P, Craig J. Consolidated criteria for reporting qualitative research (COREQ): a 32‐item checklist for interviews and focus groups. *International Journal for Quality in Health Care*. 2007. 19 (6): pp. 349–357.

**Table:**

No. item	Guide questions/description	Reported on page #
**Domain 1: Research team and reflexivity**		
*Personal characteristics*		
1. Interviewer/facilitator	Which author/s conducted the interview or focus group?	3
2. Credentials	What were the researcher's credentials? For example, PhD, MD	3
3. Occupation	What was their occupation at the time of the study?	3
4. Gender	Was the researcher male or female?	3
5. Experience and training	What experience or training did the researcher have?	3
*Relationship with participants*		
6. Relationship established	Was a relationship established prior to study commencement?	3, 13
7. Participant knowledge of the interviewer	What did the participants know about the researcher? For example, personal goals, reasons for doing the research	3, 13
8. Interviewer characteristics	What characteristics were reported about the interviewer/facilitator? For example, bias, assumptions, reasons and interests in the research topic	3–4, 13
**Domain 2: Study design**		
*Theoretical framework*		
9. Methodological orientation and theory	What methodological orientation was stated to underpin the study? For example, grounded theory, discourse analysis, ethnography, phenomenology, content analysis	3–4
*Participant selection*		
10. Sampling	How were participants selected? For example, purposive, convenience, consecutive, snowball	3
11. Method of approach	How were participants approached? For example, face‐to‐face, telephone, mail, email	3
12. Sample size	How many participants were in the study?	4
13. Non‐participation	How many people refused to participate or dropped out? Reasons?	4
*Setting*		
14. Setting of data collection	Where was the data collected? For example, home, clinic, workplace	3
15. Presence of non‐participants	Was anyone else present besides the participants and researchers?	3
16. Description of sample	What are the important characteristics of the sample? For example, demographic data, date	3
*Data collection*		
17. Interview guide	Were questions, prompts, guides provided by the authors? Was it pilot tested?	2–3
18. Repeat interviews	Were repeat interviews carried out? If yes, how many?	3
19. Audio/visual recording	Did the research use audio or visual recording to collect the data?	3
20. Field notes	Were field notes made during and/or after the interview or focus group?	3
21. Duration	What was the duration of the interviews or focus group?	3
22. Data saturation	Was data saturation discussed?	3
23. Transcripts returned	Were transcripts returned to participants for comment and/or correction?	3
**Domain 3: Analysis and findings**		
*Data analysis*		
24. Number of data coders	How many data coders coded the data?	3
25. Description of the coding tree	Did authors provide a description of the coding tree?	Table 2
26. Derivation of themes	Were themes identified in advance or derived from the data?	4
27. Software	What software, if applicable, was used to manage the data?	3
28. Participant checking	Did participants provide feedback on the findings?	4
*Reporting*		
29. Quotations presented	Were participant quotations presented to illustrate the themes/findings? Was each quotation identified? For example, participant number	4
30. Data and findings consistent	Was there consistency between the data presented and the findings?	4
31. Clarity of major themes	Were major themes clearly presented in the findings?	4–8
32. Clarity of minor themes	Is there a description of diverse cases or discussion of minor themes?	4–8

## Appendix B

### Full Interview Question Guide

**Table:**

**Domain 1 and References:** Understanding final year BDS students' stressors and well‐being [18, 19, 20, 21, 22, 23]
**Topics explored:** Dental students' well‐being during the BDS curriculum and training Students' well‐being experience(s) in their relationships with colleagues, clinical tutors and nurses Changes in the curriculum to impro‐ve student wellbeing Potential learning topics to improve personal wellbeing
Experiences
**1.1.1 Have you experienced poor mental well‐being during your BDS course?**
*YES → Can you share how frequently?*
**1.1.2 What were the stressors you experienced and how did they affect you?—Can you share how severe?**
( *Severity* : *sleep*, *mood and ability to study/socialise*)*?*
**1.1.3 Are you comfortable to share any significant events/factors relating to the BDS course that have affected your well‐being? For example, Curriculum, colleagues, staff and patients?**
*YES → How did these stressors relate to the curriculum or structure of the BDS course?*
**1.2.1 Have you ever shared your personal experiences of well‐being with your colleagues?**
*YES → What was the context?* (*what/when/why/how*)
*NO → Why not*, *how comfortable* *would you feel* *to do so? Have you shared with anyone else?*
**1.2.2/1.1.4 If you have experienced (or were to experience) poor well‐being, where did you (or would you) turn to?**
(*Colleagues*, *tutors*, *CEDARS*, etc. *—or maybe not aware of anyone?*)
(*If faculty staff not mentioned → Would you approach anyone in the faculty*, e.g., *personal tutor? Why/why not?*)
**1.1.5 Have you ever sought mental health services on or off campus (e.g., counselling, hotlines and CEDARS)?**
*YES → Are you aware of these services? Did you experience any barriers to accessing/utilising these services? How useful were these?*
*NO →* *If* *you were experiencing poor mental well‐being*, *how comfortable* *would you be* *to seek help?*
**1.1.6 Do you have any concerns about the mental health care provided by HKU?**
*Examples of potential concerns*:
*Availability*, *location and cost* *Confidentiality that may affect academic record*, *job prospects and ability to succeed in the course or career*
**1.2.3 How would you describe your overall well‐being in the context of your relationship with your colleagues?** *May discuss*: *Closeness and level of social support*, *competitiveness and tensions or conflicts*
**1.2.4 How would you describe your well‐being in the context of your relationship with your clinical tutors? Personal tutors?** *May discuss*: *Stress/pressure*, (*un*)*approachability*, *level of academic and personal support*, *and anxiety/fear*
**1.2.5 How would you describe your well‐being in the context of your relationship with your nurses?** *May discuss*: *Stress/pressure*, *poor communication/attitude*, *lack of support and tension/conflicts*
Perceptions
**1.3.1 What is your perception of the faculty's attitude towards BDS student well‐being?** *May discuss in the context of*: *Faculty's attitude to individual student well‐being* vs. *overall well‐being in the student body*, *attitude towards well‐being in the curriculum/course structure* *Examples*: *Whether well‐being is prioritised*, *whether there is enough discussion about well‐being*, *supportive environment/absence of judgement/repercussions to seeking help and enough promotion about where to seek help by faculty*
**1.3.2 Do you feel that the BDS course has given you adequate training to manage your own well‐being?**
*YES → What aspects of the BDS course and how was it helpful?*
**1.3.2 What strategies by the faculty do you think should be considered to improve dental student well‐being?** *May discuss*: *Changes in the course structure* *Flexible attendance policies for mental health leave* *In‐house counsellors at PPDH* *Survival guide* *Workshops from CEDARS/NGOs on well‐being* *Sharing from practicing dentists/recent graduates* *Mentoring/buddy system* *More support from staff/personal tutors* (*give details*)
**1.4.1 Do you think there should be more topics integrated into the BDS curriculum about well‐being?**
*YES → What kinds of topics should students learn about wellbeing? And how?*
**1.4.2 Do you think you have a professional responsibility to look after your own well‐being, in terms of how it might affect your ability to deliver patient care?**
**Domain 2 and references:** Understanding BDS students' perceptions and experiences of supporting mental well‐being of colleagues [7, 19, 20, 21, 23, 24, 25, 26, 27]
**Topics explored:** Professionalism and ability to support colleagues with poor well‐being Empathy towards colleagues Misconceptions/prejudice about mental health Potential learning topics to improve ability to provide colleague support
**2.1.1 Have you ever noticed your colleague(s) experiencing poor mental well‐being?** *YES →* *2*.*1*.*2 What were the signs you noticed?* *2*.*1*.*3 How did you react/were you able to help in any way?* *NO →* *2*.*1*.*2 Do you think there are obvious signs and symptoms of a colleague with poor mental well‐being? If so*, *what would they look like?* ***2*.*1*.*3 Have you ever intervened if a colleague started showing signs of poor mental well‐being?*** ***Are there any situations in which you would intervene if a colleague started showing signs of poor mental well‐being?*** **2.1.4 Have your colleague(s) ever disclosed to you that they are experiencing poor mental well‐being or mental illness?** *YES →* (*clarify if poor well‐being or mental illness*) *2*.*2*.*1 How did that make you feel?* *2*.*1*.*5 How did you react/were you able to help in any way?* *NO →* *2*.*2*.*1 How* *would* *you feel if it happened?* *2*.*1*.*5 How* *would* *you react?* **2.1.6 Do you think your colleagues would be likely to disclose anything to you if they were experiencing poor mental well‐being?** *YES → Why?* *NO → Why not?* **2.1.7 What about to staff in the faculty, for example, personal tutor, and so on**? *YES → Why?* *NO → Why not?* **2.1.8 Do you think dentists have a responsibility to look out for colleagues’ mental health?** *YES → Why?* *NO → Why not?* **2.2.2 If a colleague told you they had a mental illness, would that affect your relationship with them?** (E.g., *would you think less positively of the colleague*, *regard him/her as less competent*, *reliable*, etc.) **2.3.1 To what extent would you agree with the following statements? (1 = *strongly disagree*, 10 = *strongly agree*)** *2*.*3*.*1*.*1 Mental illness is a personal choice* *2*.*3*.*1*.*2 Mental illness is a sign of weakness/failure* *2*.*3*.*1*.*3 Disclosing mental illness is attention‐seeking* *2*.*3*.*1*.*4 Someone can fully recover from a mental illness*
**2.3.2 Have you witnessed any of your colleagues/tutors/nurses speaking about mental illness in a prejudiced or discriminatory way? Or in a supportive or collegial way?** *YES → How did that make you feel?* **2.4.1 Do you feel that the BDS course has given you adequate training to support colleagues with their mental well‐being?** *YES → What training did you receive? What kind of learning topics or training would you like more of?* *NO → What kind of learning topics or training would you have liked to have?*
**Domain 3 and references:** Understanding BDS students' perceptions and experiences of supporting the mental well‐being of patients [24, 28, 29].
**Topics explored:** Empathy and professionalism towards patients Awareness of relationship between mental and oral health Ability to treat patients with poor mental well‐being Potential learning topics to improve patient care with regards to mental well‐being
**3.1.1 How would you feel if your patient disclosed s/he had a mental illness?**
**3.2.1 Do you know if mental illness can affect oral health?**
*YES → In what way?*
**3.3.1 Have you come across any patients in your training who have stated that they have mental illness?**
**What about patients who, from their medical history (e.g., from current medications), you have inferred may have mental illness?**
*YES → How has interacting with them changed your perspective?*
**3.3.2 How comfortable would you feel with treating patients with mental illness?**
**3.3.3 Do you feel like the BDS training adequately covers treating dental patients with mental illness?**
*YES → Why?*
*NO → Why not?*
**3.4.1 Would you find training on supporting the mental well‐being of patients helpful?**
*YES → What kind of topics would you like such training to cover?*
*NO → Why not?*
**3.4.2 To what extent do you think that understanding mental illness will make you a better dentist?**
**Other open‐ended questions**
Before we end the interview, do you have anything else to add?
